# A Preclinical Systematic Review and Meta-Analysis of Astragaloside IV for Myocardial Ischemia/Reperfusion Injury

**DOI:** 10.3389/fphys.2018.00795

**Published:** 2018-07-03

**Authors:** Qun Zheng, Jia-Zhen Zhu, Xiao-Yi Bao, Peng-Chong Zhu, Qiang Tong, Yue-Yue Huang, Qi-Hao Zhang, Ke-Jian Zhang, Guo-Qing Zheng, Yan Wang

**Affiliations:** ^1^Department of Cardiology, The Second Affiliated Hospital and Yuying Children's Hospital of Wenzhou Medical University, Wenzhou, China; ^2^Department of Neurology, The Second Affiliated Hospital and Yuying Children's Hospital of Wenzhou Medical University, Wenzhou, China

**Keywords:** Astragaloside IV, myocardial ischemia/reperfusion injury, *Astragalus membranaceus Bunge*, preclinical systematic review, meta-analysis

## Abstract

Astragaloside IV (AS-IV), the major pharmacological extract from *Astragalus membranaceus Bunge*, possesses a variety of biological activities in the cardiovascular systems. Here, we aimed to evaluate preclinical evidence and possible mechanism of AS-IV for animal models of myocardial ischemia/reperfusion (I/R) injury. Studies of AS-IV in animal models with myocardial I/R injury were identified from 6 databases from inception to May, 2018. The methodological quality was assessed by using CAMARADES 10-item checklist. All the data were analyzed using Rev-Man 5.3 software. As a result, 22 studies with 484 animals were identified. The quality score of studies ranged from 3 to 6 points. Meta-analyses showed AS-IV can significantly decrease the myocardial infarct size and left ventricular ejection fraction, and increase shortening fraction compared with control group (*P* < 0.01). Significant decreasing of cardiac enzymes and cardiac troponin and increasing of decline degree in ST-segment were reported in one study each (*P* < 0.05). Additionally, the possible mechanisms of AS-IV for myocardial I/R injury are promoting angiogenesis, improving the circulation, antioxidant, anti-inflammatory and anti-apoptosis. Thus, AS-IV is a potential cardioprotective candidate for further clinical trials of myocardial infarction.

## Introduction

Acute myocardial infarction (AMI) was one of the leading causes of morbidity and mortality worldwide (Dariush et al., 2016). Acute interruption of coronary artery led to cardiomyocyte ischaemia and apoptosis (Luo et al., 2015). Invasive vascular reconstructions such as percutaneous coronary intervention and coronary artery bypass grafting can improve coronary perfusion (Richard, 2011), and thus they were widely adopted after weighing the risks of invasive diagnostics and the benefits in terms of diagnostic accuracy, risk stratification and assessment of the risks related to revascularization (Damman et al., 2015). Although treatment is usually directed at prompt restoration of flow in the occluded artery, reperfusion may trigger further injury beyond that induced by ischaemia alone (Maria et al., 2016). Such ischaemia/reperfusion (I/R) injury can markedly reduce the benefits of reperfusion therapies employed in myocardial infarction (MI) (Yellon and Hausenloy, 2007).

**Figure 1:**
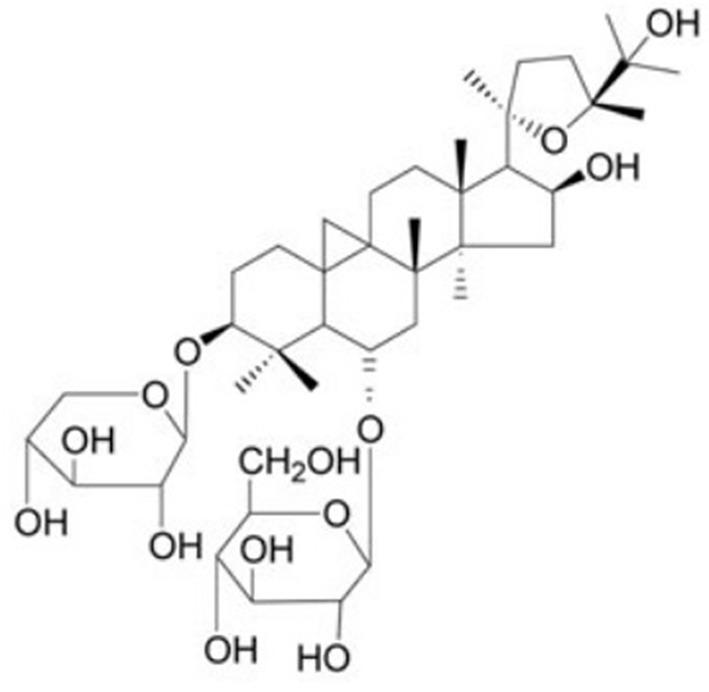
Chemical structures of astragaloside IV.

Astragaloside IV (AS-IV) (Figure 1) is one of the major and active components isolated from *Astragalus membranaceus Bunge* for tonifying *Qi*, and is a lanolin alcohol-shaped tetracyclic triterpenoid saponin with high polarity. Recent experimental studies (Ren et al., 2013; Li et al., 2017) demonstrated that AS-IV had pleiotropic anti-ischemic properties against focal cerebral ischemia/reperfusion injury, cardiovascular disease, pulmonary disease, liver fibrosis and diabetic nephropathy. AS-IV has multiple pharmacologic effects, including regulation of the calcium balance, antioxidative stress, anti-inflammatory, antiapoptosis antifibrotic, antidiabetes, immunoregulation, and cardioprotective effect via numerous signaling pathways (Schmidt et al., 2007; Ren et al., 2013; Li et al., 2017). In addition, systemic review of animal studies plays a critical role in drug development and the clarification of physiological and pathological mechanisms of clinical research (Rob et al., 2014). Thus, we conducted a preclinical systematic review to evaluate the effectiveness and the mechanisms of AS-IV for experimental MI.

## Methods

### Database and literature search strategies

Preferred Reporting Items for Systematic Review and Meta-Analyses (PRISMA)statement was followed (Stewart et al., 2015). Experimental studies assessing the effects of AS-IV in animal models of MI were identified from PubMed, EMBASE, Science Direct, Web of Science, wanfang data Information Site, Chinese National Knowledge Infrastructure (CNKI), and VIP information database by searching for all published articles from inception to May, 2018. The following key words were used: “astragaloside (MeSH Terms) OR astragaloside (Title/Abstract)” AND “myocardial infarction OR myocardial ischemia OR myocardial ischemia/reperfusion injury OR myocardial I/R injury,” Moreover, reference lists of potential articles were searched for additive studies.

### Eligibility criteria

We included studies of the effect of AS-IV in animal models of MI. To prevent bias, inclusion criteria were prespecified as follows: (1) experimental MI was induced by transient left anterior descending coronary artery (LAD) ligation or permanent LAD ligation or isoproterenol (ISO); (2) the treatment group was received AS-IV as monotherapy in any dose. Interventions for control group were isasteric and non-functional liquid (normal saline) or no treatment; (3) the primary outcome measures were MI size and/or left ventricular ejection fraction (LVEF) and/or shortening fraction (FS) and/or the level of ST-segment depression cardiac and/or enzymes and/or cardiac troponin T (cTnT) and/or cardiac troponin I (cTnI). The second outcome measures were mechanisms of AS-IV for myocardial I/R injury. Prespecified exclusion criteria were treatment with AS-IV conjunction with other compounds or AS-IV based prescriptions, non-myocardial ischemia model, no control group, duplicate publications, and no available data.

### Data extraction

Two independent authors extracted the following details from included studies: (1) publication year and the first author's name, model of MI (transient or permanent); (2) the characteristics of animals used including animal number, species, sex, weight, age, and any comorbidity; (3) model of myocardial I/R, and the anesthesia methods for model preparation; (4) the information of treatment group, including therapeutic drug dosage, method of administration, duration of treatment, and the same information of control group; (5) mean value and standard deviation of outcomes. If outcomes were performed at different time points, only the final test was included. If the experimental group of animals received various doses of the drug therapy, only the data of highest dose of the drug was included. If the data for meta-analysis were missing or only expressed graphically, we tried to contact the authors for further information, and where a response was not received, we measured data from the graphs using digital ruler software or exclude. For each comparison, we extracted data of mean value and standard deviation from each experimental and control group of every study.

### Quality assessment

We evaluated the methodological quality of the included studies using the collaborative Evidence-Based Complementary and Alternative Medicine approach to meta-analysis and review of animal data in experimental infarction (CAMARADES) 10-item quality checklist (Malcolm et al., 2004) with minor modification (Yu L. J. et al., 2017). One point was awarded for each of (1) publication in a peer-reviewed journal; (2) statement of temperature control; (3) random allocation to groups; (4) allocation concealment; (5) blinded assessment of outcome; (6) use of anesthetic without significant intrinsic cardioprotective activity; (7) appropriate animal model (aged, diabetic, or hypertensive); (8) sample size calculation; (9) compliance with animal welfare regulations; (10) statement of potential conflict of interests.

### Statistical analysis

All CI were considered as continuous data, and then an estimate of the combined effect sizes utilizing mean difference (MD) or standard mean difference (SMD) with the random effects model was given. In the present meta-analysis, the results using the random effects model were presented because heterogeneity between multistudies has to be taken into account. *I*^2^ statistic was used to assess heterogeneity. The significance of differences among groups was assessed by partitioning heterogeneity and by using the *X*^2^ distribution with degrees of freedom (df), where equals the number of groups. Probability values 0.05 were considered significant. All analyses were performed with Revman version 5.3 by the Cochrane Collaboration.

## Results

### Study inclusion

**Figure 2:**
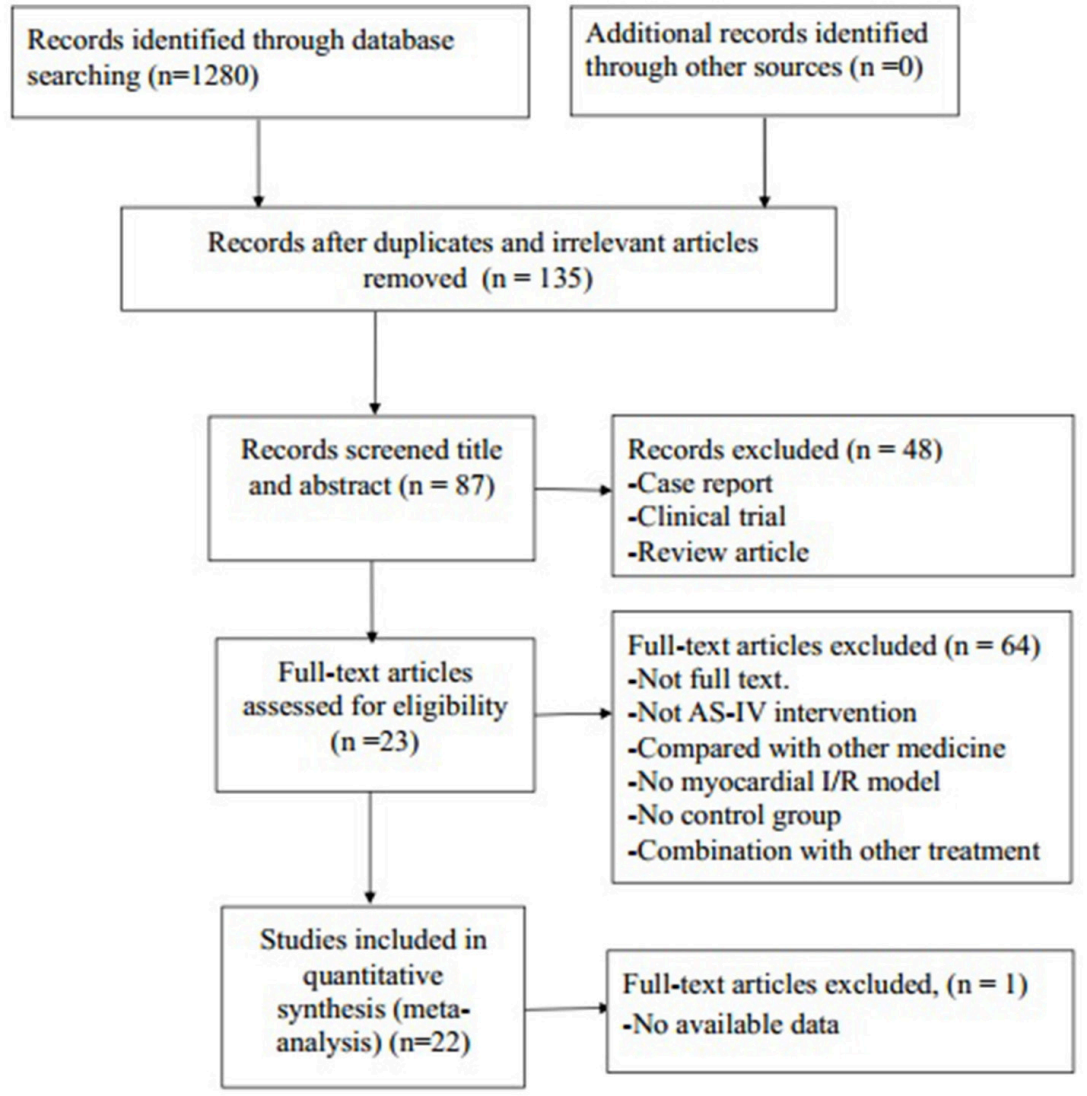
Summary of the process for identifying candidate studies.

We identified 1,280 potentially relevant articles, of which 1,045 were reduplicated and irrelevant articles. Through screening titles and abstracts, 48 papers were excluded with at least one of following reasons: (1) clinical trial; (2) case report; (3) review article. By reading the fulltext of the remaining 87 articles, 65 articles were excluded because of at least one of the following reasons: (1) not full text; (2) not AS-IV intervention; (3) no available data; (4) compared with traditional Chinese medicine; (5) no MI/R model; (6) no control group; (7) no available data. Ultimately, 22 eligible articles (Zhang et al., 2005, 2014; Zhao et al., 2009; Guan et al., 2010; Liu et al., 2010; Wang et al., 2012, 2018; Cui et al., 2013; Gong and Sun, 2013; Tu et al., 2013; He et al., 2014; Liu and Yi, 2014; Qu et al., 2014; Huang et al., 2015; Lu et al., 2015; Ma and Wang, 2015; Sun et al., 2015; Yu et al., 2015; Cheng et al., 2016; Li and Yang, 2016; Li et al., 2016; Yu J. et al., 2017) were identified (Figure 2).

### Study characteristics

A total of 484 animals were included in the 22 studies, of which 234 were in the experimental group and 230 were in the control group. Eight studies (Zhang et al., 2005; Wang et al., 2012; Gong and Sun, 2013; Tu et al., 2013; Lu et al., 2015; Yu et al., 2015; Cheng et al., 2016; Yu J. et al., 2017) were published in English and 14 studies (Zhao et al., 2009; Guan et al., 2010; Liu et al., 2010; Cui et al., 2013; He et al., 2014; Liu and Yi, 2014; Qu et al., 2014; Zhang et al., 2014; Huang et al., 2015; Ma and Wang, 2015; Sun et al., 2015; Li and Yang, 2016; Li et al., 2016; Wang et al., 2018) were published in Chinese between 2005 and 2018. Twelve studies (Cui et al., 2013; Gong and Sun, 2013; Tu et al., 2013; Liu and Yi, 2014; Qu et al., 2014; Zhang et al., 2014; Huang et al., 2015; Lu et al., 2015; Ma and Wang, 2015; Sun et al., 2015; Cheng et al., 2016; Wang et al., 2018) used Sprague Dawley rats; 6 studies (Zhao et al., 2009; Guan et al., 2010; Wang et al., 2012; He et al., 2014; Yu et al., 2015; Yu J. et al., 2017) used Wistar rats; 1 study (Li et al., 2016) used unknown breed rats; 1 study (Li et al., 2016) used C57/BL6 mice; 1 study (Liu et al., 2010) used Beagle dogs and 1 study (Zhang et al., 2005) used Mongrel dogs. Eighteen studies (Zhang et al., 2005, 2014; Zhao et al., 2009; Guan et al., 2010; Wang et al., 2012, 2018; Cui et al., 2013; Tu et al., 2013; He et al., 2014; Liu and Yi, 2014; Qu et al., 2014; Huang et al., 2015; Lu et al., 2015; Ma and Wang, 2015; Yu et al., 2015; Cheng et al., 2016; Li and Yang, 2016; Yu J. et al., 2017) used male animals, two studies (Liu et al., 2010; Cui et al., 2013) used both female and male rats, and two studies (Sun et al., 2015; Li et al., 2016) did not mention gender of animals. All studies reported that myocardial I/R models were produced by ligation of the LAD. To induce anesthesia, 3 studies (Wang et al., 2012; Gong and Sun, 2013; Ma and Wang, 2015) used chloral hydrate; 13 studies (Zhang et al., 2005, 2014; Cui et al., 2013; Tu et al., 2013; He et al., 2014; Liu and Yi, 2014; Qu et al., 2014; Huang et al., 2015; Yu et al., 2015; Cheng et al., 2016; Li and Yang, 2016; Li et al., 2016; Yu J. et al., 2017) used pentobarbital sodium; 3 studies (Guan et al., 2010; Lu et al., 2015; Wang et al., 2018) used urethane; and anesthetic was not mentioned in remaining 3 studies (Zhao et al., 2009; Huang et al., 2015; Sun et al., 2015). Among the dose use of AS-IV, 1 study (Gong and Sun, 2013) used 100 mg∙kg^−1^∙d^−1^; 2 study (Lu et al., 2015; Wang et al., 2018) used 80 mg∙kg^−1^∙d^−1^; 1 study (Cheng et al., 2016) used 50 mg∙kg^−1^∙d^−1^; 1 study (Wang et al., 2012) used 40 mg∙kg^−1^∙d^−1^; 10 studies (Cui et al., 2013; Tu et al., 2013; He et al., 2014; Zhang et al., 2014; Ma and Wang, 2015; Sun et al., 2015; Yu et al., 2015; Li and Yang, 2016; Yu J. et al., 2017) utilized 10 mg∙kg^−1^∙d^−1^; 1 study (Guan et al., 2010) used 5 mg∙kg^−1^∙d^−1^; 2 studies (Qu et al., 2014; Huang et al., 2015) adopted 4 mg∙kg^−1^∙d^−1^; 1 study (Li et al., 2016) used 2 mg∙kg^−1^∙d^−1^; 1 study (Zhang et al., 2005) used 1.5 mg∙kg^−1^∙d^−1^; 1 study (Liu and Yi, 2014) used 50 uM/kg; the remaining 2 studies (Zhao et al., 2009; Liu et al., 2010) used 1 mg∙kg^−1^. Ten studies (Zhang et al., 2005, 2014; Tu et al., 2013; He et al., 2014; Liu and Yi, 2014; Lu et al., 2015; Cheng et al., 2016; Li et al., 2016; Yu J. et al., 2017; Wang et al., 2018) utilized MI size as outcome measure, and myocardial cell apoptosis rate in 5 studies (Zhao et al., 2009; Liu and Yi, 2014; Lu et al., 2015; Ma and Wang, 2015; Sun et al., 2015). The level of ST-segment depression was reported in 1 study (Liu et al., 2010), the LVEF in 5 studies (Wang et al., 2012; Zhang et al., 2014; Cheng et al., 2016; Li and Yang, 2016; Li et al., 2016) and FS in 6 studies (Zhao et al., 2009; Wang et al., 2012; Zhang et al., 2014; Cheng et al., 2016; Li and Yang, 2016; Li et al., 2016). Lactate dehydrogenase (LDH) was reported in 2 studies (Guan et al., 2010; Qu et al., 2014) creatine kinase (CK) in 1 study (Qu et al., 2014), cTnT in 1 study (Tu et al., 2013); cTnI in 1 study (Wang et al., 2018), superoxide dismutase (SOD) in 1 study (Guan et al., 2010), malondialdehyde (MDA) in 1 study (Guan et al., 2010), hypoxia-inducible factor 1-α (HIF-1α) 1 study (Yu J. et al., 2017), caspase-3 in 3 studies (Liu and Yi, 2014; Lu et al., 2015; Ma and Wang, 2015), calcium-sensing receptor vascular in 1 study (Wang et al., 2018), endothelium growth factor (VEGF) in 2 studies (Yu et al., 2015; Li et al., 2016) tumor necrosis factor-α (TNF-α) in 1 study (Lu et al., 2015), nuclear factor κB (NF-κB) in 3 study (Tu et al., 2013; Lu et al., 2015; Cheng et al., 2016), NO in 1 study (Guan et al., 2010), coronary blood flow (CBF) in 2 studies (Zhang et al., 2005; Liu et al., 2010), microvessel density (MVD) in 3 studies (Yu et al., 2015; Li and Yang, 2016; Li et al., 2016), and Bax and Bcl-2 in 7 studies (Zhao et al., 2009; Tu et al., 2013; Liu and Yi, 2014; Lu et al., 2015; Ma and Wang, 2015; Cheng et al., 2016; Wang et al., 2018). The characteristics of the 22 included studies were summarized in detail in Table 1.

**Table 1:** Characteristics of the 22 included studies.

**Study (years)**	**Species (Sex, *n* = experimental /control group)**	**Weight**	**Model (method)**	**Anesthetic**	**Treatment group (method to astragalosides)**	**Control group**	**Outcome index (time)**	**Intergroup differences**
Zhang et al., 2005	Mongrel dogs (male, 6/6)	12–15 kg	The LAD was ligated for 180 min	Pentobarbital sodium (30 mg/kg)	IntravenousAS-IV (1.5 mg/kg) 30 min before the ligation	Intravenous isasteric no-function solvent	1. Myocardial infarct size; 2. CPK; 3.CBF;	1. *P* < 0.05; 2. *P* < 0.05; 3. *P* < 0.05;
Zhao et al., 2009	Wistar rats (10/10)	215–240 g	Ligation of the LAD	Not mention	Intravenous injection AS-IV (1.0 mg/kg) once a day for 14 days after coronary ligation for 3 weeks	Intravenous equal volumes of normal saline	1. Myocardial cell apoptosis rate; 2. FS; 3. +dp/dt; 4. -dp/dt; 5. LVIDd; 6. LVIDs; 7. LVSP; 8. LVEDP; 9. Wall stress; 10. Tibial length; 11. Body weight; 12. Bax;	1. *P* < 0. 001; 2. *P* < 0.01; 3. *P* < 0.001; 4. *P* < 0.05; 5. *P* < 0.001; 6. *P* > 0.05; 7. *P* < 0.01; 8. *P* < 0.05; 9. *P* > 0.05; 10. *P* > 0.05; 11. *P* < 0.001; 12. *P* < 0.001;
Li et al., 2010	Beagle dogs (male and female, 5/5)	8–12 kg	Ligation of LAD	3% pentobarbital sodium (30 mg/kg)	Intravenous AS-IV (1 mg/kg) after AMI model established for 15 min	Intravenous isasteric placebo after AMI model established for 15 min	1. ΔST-E; 2. CBF; 3. BP; 4. LVEDP; 5. CI;	1. *P* < 0.01; 2. *P* < 0.05; 3. *P* > 0.05; 4. *P* > 0.05; 5. *P* > 0.05;
Carol et al., 2010	Wistar rats (male, 6/6)	200–250 g	Ligation of LAD for 30 min then reperfusion	Urethane (1 g/kg)	Intravenous AS-IV (5 mg/kg)	Intravenous isasteric normal saline (2 mg/kg)	1. LDH; 2. SOD; 3. MDA; 4. NO; 5. PKCε;	1. *P* < 0.05; 2. *P* < 0.05; 3. *P* < 0.05; 4. *P* < 0.01 5. *P* < 0.05;
Wang et al., 2012	Wistar rats (male, 10/9)	250–300 g	Ligation of the LAD	Chloral hydrate (300 mg/kg)	Treated with AS-IV (40 mg/kg)	Treated with equal volumes of distilled water	1. LVEF; 2. FS; 3. LVEDV; 4. LVESV; 5. LVDd; 6. LVDs; 7. LVPWd; 8. LVPWs; 9. SERCA activity; 10. SERCA mRNA; 11. PLB mRNA; 12. PLB; 13. P-PLB; 14. P-PLB/PLB; SERCA;	1. *P* < 0.01; 2. *P* < 0.05; 3. *P* < 0.01; 4. *P* < 0.05; 5. *P* < 0.01; 6. *P* < 0.01; 7. *P* < 0.01 8. *P* < 0.01; 9. *P* < 0.05; 10. *P* < 0.05; 11. *P* < 0.05; 12. *P* > 0.05; 13. *P* > 0.05; 14. *P* > 0.05;
Tu et al., 2013	SD rats (male, 6/6)	240–260 g	Ligation of LAD for 30 min then reperfusion for 90 min	2% pentobarbital sodium (60 mg/kg)	Gavaged with AS-IV (10 mg/kg) in saline 90 min before ischemia	Gavaged with isasteric normal at 1 mL/kg	1. AAR/LV; 2. Infarct area/aar; 3. AAR/LV; 4. MBF; 5. HR; 6. LVSP; 7. +dp/dt_max_; 8. LVEDP; 9. LVDP; 10. -dp/dt_max_; 11. cTnI; 12. ATP/ADP(I); 13. ATP/AMP(I); 14. ATP/ADP(I/R); 15. ATP/AMP(I/R); 16. ATP-5D(I); 17. ATP 5D mRNA(I); 18. P-MLC2(I); 19. ATP-5D(I/R); 20. ATP 5D mRNA(I/R); 21. P-MLC2(I/R); 22. Bax/Bcl-2(I/R);	1. *P* > 0.05; 2. *P* < 0.05; 3. *P* > 0.05; 4. *P* > 0.05; 5. *P* < 0.05; 6. *P* < 0.05; 7. *P* < 0.05; 8. *P* < 0.05; 9. *P* < 0.05; 10. *P* < 0.05; 11. *P* < 0.05; 12. *P* < 0.05; 13. *P* < 0.05; 14. *P* < 0.05; 15. *P* < 0.05; 16. *P* < 0.05; 17. *P* < 0.05; 18. *P* < 0.05; 19. *P* < 0.05; 20. *P* < 0.05; 21. *P* < 0.05; 22. *P* < 0.05;
Cui et al., 2013	SD rats (male, 16/15)	200–250 g	Ligation of LAD	3% pentobarbital sodium (30 mg/kg)	Gavaged with AS-IV (10 mg/kg) after model established 1 day, once a day, for 40 days	Gavaged with isasteric normal saline after model established 1 day, once a day, for 40 days	1. LVEDP; 2. LVSP; 3. +dp/dt_max;_; 4. –dp /dt_max_; 5. AngII; 6. ALD; 7. ANP;	1. *P* < 0.01; 2. *P* < 0.01; 3. *P* < 0.01; 4. *P* < 0.01; 5. *P* < 0.01; 6. *P* < 0.01; 7. *P* < 0.01;
Gong and Sun, 2013	SD rats (male and female, 10/9)	250–320 g	Ligation of the LAD for 30 min then reperfusion for 40 min	10% chloral hydrate (0.3 ml/100 g)	Treated with AS-IV (100 mg/kg) twice a day, for 7 days	Treated with saline in the same way, twice a day, 7 days	1. Intracecelluar the concentration of free Ca^2+^;	1. *P* < 0.01;
Qu et al., 2014	SD rats (male, 4/4)	250–350 g	Ligation of LAD for 30 min then reperfusion	Pentobarbital sodium	Tail intravenous AS - IV (4 mg/kg) after I/R model established,3 times a week for 2 weeks	Tail intravenous isasteric normal saline after I/R model established,3 times a week for 2 weeks	1. LDH; 2. CK; 3. AST;	1. *P* < 0.05; 2. *P* < 0.05; 3. *P* < 0.05;
He et al., 2014	Wistar rats (male 10/10)	250–350 g	The LAD was ligated for 30 min, then reperfusion for 120 min	Phenobarbital (100 mg/kg)	Intravenous AS-IV (10 mg/kg)	Intravenous isasteric normal	1. Myocardial infarct size; 2. LVDP;	1. *P* < 0.05; 2. *P* < 0.05;
Liu and Yi, 2014	SD rats (male, 8/8)	220–240 g	The LAD was ligated for 90 min, then reperfusion for 60 min	pentobarbital sodium (25 mg/kg)	Intravenous AS-IV (50 uM/kg)	Intravenous isasteric normal	1. Myocardial infarct size; 2. LVDP; 3. Myocardial cell apoptosis rate; 4. Caspase-3; 5. Bcl-2; 6. LDH activity;	1. *P* < 0.05; 2. *P* < 0.05; 3. *P* < 0.05; 4. *P* < 0.05; 5. *P* < 0.05; 6. *P* < 0.05;
Zhang et al., 2014	SD rats (male, 15/15)	250–350 g	Ligation of LAD for 30 min then reperfusion for 120 min	Pentobarbital sodium (100 mg/kg)	Intravenous AS-IV (10 mg/kg) 1 ml 5 min earlier before reperfusion	Intravenous isasteric and non-functional liquid 5 min earlier before reperfusion	1. Myocardial iInfarct size(I/R); 2. LVEF; 3. P-Akt/Akt; 4. P-mTOR/mTOR; 5. LVSP; 6. LVEDP; 7. FS;	1. *P* < 0.05; 2. *P* < 0.05; 3. *P* < 0.05; 4. *P* < 0.05; 5. *P* < 0.05; 6. *P* < 0.05; 7. *P* < 0.05;
Huang et al., 2015	SD rats (male, 4/4)	250–350 g	Ligation of LAD	Not mention	Tail intravenous AS - IV (4 mg/kg) after I/R model established, 3 times a week for 2 weeks	Tail intravenous isasteric and non-functional liquid after I/R model established, 3 times a week for 2 weeks	1. Size of inflammatory cell infiltration; 2. Beclin protein;	1. *P* < 0.05; 2. *P* < 0.05;
Ma and Wang, 2015	SD rats (male, 31/30)	240-360 g	Ligation of LAD for 10 min then reperfusion for 60 min	10% chloral hydrate (5 mg/kg)	Gavaged with AS-IV (10 mg/kg) for 2 weeks before model established	Gavaged with saline (2 ml/kg/d) for 2 weeks before model established	1. Myocardial cell apoptosis rate; 2. Bax; 3. Bcl-2; 4. caspase-3;	1. *P* < 0.05; 2. *P* < 0.05; 3. *P* < 0.05; 4. *P* < 0.05;
Sun et al., 2015	SD rats (gender is not mentioned, 10/10)	240–260 g	Ligation of LAD	Not mention	Gavaged with AS-IV (10 mg/kg) after model was established 1 day, once a day for 2 weeks	Gavaged with isasteric normal saline after model established 1 day, once a day for 2 weeks	1. Myocardial cell apoptosis rate; 2. Intracecelluar the concentration of free Ca2+; 3. GRP78; 4. Caspase-1;	1. *P* < 0.05; 2. *P* < 0.05; 3. *P* < 0.05; 4. *P* < 0.05;
Yu et al., 2015	Wistar rats (male, 8/8)	250–300 g	Ligation of LAD	1% pentobarbital sodium (40 mg/kg)	24 h after the surgery,via intraperitoneal injection for 4 weeks (10 mg/kg/d)	Normal saline of equal volume was administered via i.p. Injection for 4 weeks	1. Myocardial infarct size; 2. VEGF; 3. BFGF; 4. MVD;	1. *P* < 0.05; 2. *P* < 0.05; 3. *P* < 0.05; 4. *P* < 0.05;
Lu et al., 2015	SD rats (male, 10/10)	280–300 g	The LAD was ligated for 30 min, then reperfusion for 120 min	20% urethane (0.3 ml/100 g, intraperitoneal)	Gavaged with AS-IV (80 mg/kg) suspended in 0.5% sodium carboxymethylcellulose daily for 7 days	Gavaged with 0.5% sodium carboxymethylcellulose for 7 days	1. Myocardial infarct size; 2. Myocardial cell apoptosis rate; 3. TNF-α; 4. Caspase-3; 5. Bcl-2; 6. Bax; TLR4 7. mRNA; 8. TLR4; 9. NF-κB; 10. IL-1β;	1. *P* < 0.01; 2. *P* < 0.01; 3. *P* < 0.01; 4. *P* < 0.01; 5. *P* < 0.01; 6. *P* < 0.01; 7. *P* < 0.01; 8. *P* < 0.01; 9. *P* < 0.01; 10. *P* < 0.01;
Li et al., 2016	Rats (24/24)	22–28 g	Ligation of LAD	Pentobarbital sodium(20 g/L)	Intravenous AS-IV (2 mg/kg/d) for 21 days	Intravenous isasteric and non-functional liquid for 21 days	1. Myocardial infarct size; 2. FS; 3. LVEF; 4. MVD; 5. VEGF; 6. HIF-1α;	1. *P* < 0.05; 2. *P* < 0.05; 3. *P* < 0.05; 4. *P* < 0.01; 5. *P* < 0.01; 6. *P* < 0.01; 7. *P* < 0.01;
Li et al., 2016	C57/BL6 rats (male, 12/12)	23–25 g	Ligation of LAD	1% pentobarbital sodium (70 mg/kg)	Intravenous AS-IV (10 mg/kg) for 2 weeks after coronary ligation	Intravenous isasteric normal saline for 2 weeks after coronary ligation	1. LVEF; 2. FS; 3. LVID 4. LVW/BW; 5. MVD;	1. *P* < 0.05; 2. *P* < 0.05; 3. *P* < 0.05; 4. *P* < 0.05; 5. *P* < 0.05;
Cheng et al., 2016	SD rats (male, 15/15)	230–270 g	Ligation of LAD	pentobarbital sodium (50 mg/kg)	Intravenous AS-IV (50 mg/kg/d) for 14 days before model established	Intravenous isasteric normal saline for 14 days before model established	1. Myocardial infarct size; 2. LVEF; 3. FS; 4. Bcl-2; 5. Bax; 6. TLR4; 7. NF-κB;	1. *P* < 0.05; 2. *P* < 0.05; 3. *P* < 0.05; 4. *P* < 0.05; 5. *P* < 0.05; 6. *P* < 0.05; 7. *P* < 0.05;
Yu J. et al., 2017	Wistar rats (male, 15/15)	220–280 g	Ligation of LAD	1% pentobarbital sodium (40 mg/kg)	Intravenous AS-IV (10 mg/kg/d) for 28 days before model established	Intravenous isasteric normal saline for 28 days before model established	1. Gross cardiac morphology; 2. HIF-1α; 3. Notch1; 4. Jagged1;	1. *P* < 0.05; 2. *P* < 0.05; 3. *P* < 0.05; 4. *P* < 0.05;
Wang et al., 2018	SD rats (male, 8/8)	220–250 g	The LAD was ligated for 30 min, then reperfusion for 120 min	20% Urethane	Intravenous AS-IV (80 mg/kg)	Intravenous nothing	1. Myocardial infarct size; 2. cTnI; 3. Bcl-2; 4. Bax; 5. CaSR;	1. *P* < 0.05; 2. *P* < 0.05; 3. *P* < 0.05; 4. *P* < 0.05; 5. *P* < 0.05;

*AI, apoptotic index; NS, normal saline; AS, astragaloside; AS-IV, astragaloside-IV; AMI, acute myocardial infarction; AAR, area at risk; LAD, the left anterior descending coronary artery; SD, Sprague-Dawley; SOD, superoxide dismutase, MDA, malondialdehyde; CK, creatine kinase; LDH, lactate dehydrogenase; cTnT, cardiac troponin T; cTnI, cardiac troponin I; MVD, microvessel density; TNF-α, tumor necrosis factor-α; CBF, coronary blood flow; CI, cardiac index; BP, blood pressure; HR, heart rate; RPP, rate-pressure product; VEGF, vascular endothelial growth fartor; FS, shortening fraction; ALD, aldosterone; ANP, atrial natriuretic peptide; Ang, Angiotensinogen; LVIDd, left ventricular internal diameter in diastole; LVIDs, left ventricular internal diameter in systole; LVEF, left ventricular ejection fraction; LVSP, left ventricular systolic pressure; LVEDP, left ventricular end-diastolic pressure; LVID, left ventricular internal dimension; LVESV, left ventricular end-systolic volume; LVEDV, left ventricular end-diastolic volume; LVDd, left ventricular end-diastolic dimensions; LVDs, left ventricular endsystolic dimensions; LVPW, left Ventricular Posterior Wall; LVPWd, left ventricular posterior wall thickness at end-diastolic; LVPWs, left ventricular posterior wall thickness at end-systolic; MAP, mean arterial pressure; LVM, left ventricular mass; AST, aspartate amino transferase; BFGF, basic fibroblast growth factor; CPK, creatine phosphokinase; MBF, myocardial blood flow; HIF-1α, hypoxia-inducible factor 1-α; CaSR, calcium-sensing receptor; NF-κB, nuclear factor κB*.

### Study quality

All the included records were peer reviewed publications and all animals were allocated randomly to treatment group and control group; however, no study reported a sample size calculation, blinded induction of model and blinding their assessment of outcome. Nine studies (Zhao et al., 2009; Wang et al., 2012; Tu et al., 2013; Lu et al., 2015; Yu et al., 2015; Cheng et al., 2016; Li and Yang, 2016; Li et al., 2016; Yu J. et al., 2017) reported control of temperature. Nine studies (Zhang et al., 2005, 2014; Zhao et al., 2009; Wang et al., 2012; Tu et al., 2013; Lu et al., 2015; Yu et al., 2015; Cheng et al., 2016; Yu J. et al., 2017) reported a compliance with animal welfare regulations, and 6 studies (Zhao et al., 2009; Wang et al., 2012; Lu et al., 2015; Cheng et al., 2016; Li and Yang, 2016; Yu J. et al., 2017) mentioned a statement of potential conflict of interests. Five studies (Liu et al., 2010; Qu et al., 2014; Huang et al., 2015; Sun et al., 2015; Yu et al., 2015) described appropriate animal models (aged, diabetic, or hypertensive). All studies except two studies (Zhao et al., 2009; Sun et al., 2015) used an anesthetic without intrinsic cardioprotective properties. To summarize, the quality score of included studies ranges from 3 to 6. Of which, 10 studies (Zhang et al., 2005; Guan et al., 2010; Cui et al., 2013; Gong and Sun, 2013; He et al., 2014; Liu and Yi, 2014; Huang et al., 2015; Ma and Wang, 2015; Sun et al., 2015; Wang et al., 2018) got 3 points; 5 studies (Liu et al., 2010; Tu et al., 2013; Qu et al., 2014; Zhang et al., 2014; Li et al., 2016) got 4 points; 3 studies (Zhao et al., 2009; Yu et al., 2015; Li and Yang, 2016) got 5 points; and 4 studies (Wang et al., 2012; Lu et al., 2015; Cheng et al., 2016; Yu J. et al., 2017) got 6 points. The methodological quality of each study was summarized in Table 2.

**Table 2:** Risk of bias of the included studies.

**Study**	**A**	**B**	**C**	**D**	**E**	**F**	**G**	**H**	**I**	**J**	**Total**
Zhang et al., 2014	√		√			√			√		4
Zhang et al., 2005	√					√			√		3
Zhao et al., 2009	√	√	√						√	√	5
Liu et al., 2010	√		√			√	√				4
Guan et al., 2010	√		√			√					3
Wang et al., 2012	√	√	√			√			√	√	6
Cui et al., 2013	√		√			√					3
Tu et al., 2013	√	√				√			√		4
Gong and Sun, 2013	√		√			√					3
Qu et al., 2014	√		√			√	√				4
He et al., 2014	√		√			√					3
Liu and Yi, 2014	√		√			√					3
Huang et al., 2015	√		√				√				3
Ma and Wang, 2015	√		√			√					3
Sun et al., 2015	√		√				√				3
Yu et al., 2015	√	√				√	√		√		5
Lu et al., 2015	√	√	√			√			√	√	6
Li and Yang, 2016	√	√	√			√					4
Li and Yang, 2016	√	√	√			√				√	5
Cheng et al., 2016	√	√	√			√			√	√	6
Yu J. et al., 2017	√	√	√			√			√	√	6
Wang et al., 2018	√		√			√					3

*Studies fulfilling the criteria of: A, peer reviewed publication; B, control of temperature; C, random allocation to treatment or control; D, blinded induction of model; E, blinded assessment of outcome; F, use of anesthetic without significant intrinsic cardioprotective activity; G, appropriate animal model (aged, diabetic, or hypertensive); H, sample size calculation; I, compliance with animal welfare regulations; J, statement of potential conflict of interest*.

### Effectiveness

#### MI size

**Figure 3:**
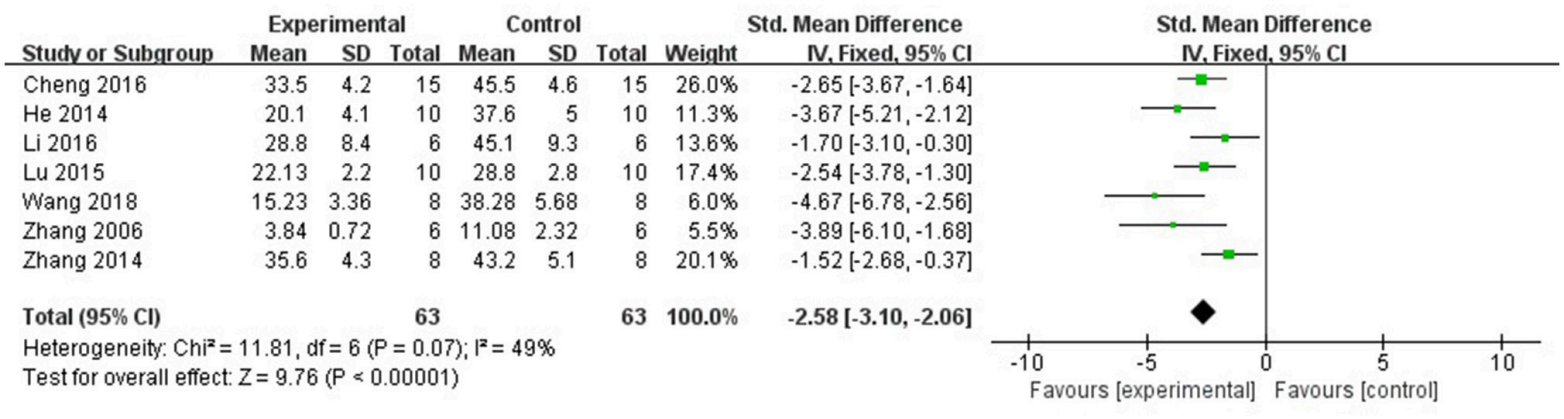
The forest plot: effects of astragaloside IV for decreasing the myocardial infarction size compared with control group.

Ten studies utilized MI size (Zhang et al., 2005, 2014; Tu et al., 2013; He et al., 2014; Liu and Yi, 2014; Lu et al., 2015; Cheng et al., 2016; Li et al., 2016; Yu J. et al., 2017; Wang et al., 2018) as outcome measure. All of them showed significant effect of AS-IV for decreasing the MI size (*P* < 0.05). However, 1 study (Liu and Yi, 2014) that MI experimental model was induced by blocking LAD for 90 vs. 30 min in other studies; 1 study (Tu et al., 2013) showed that the data of MI size is inconsistent between figure and form; 1 study (Yu J. et al., 2017) only observe the gross cardiac morphology without measuring data. Meta-analysis of 7 studies (Zhang et al., 2005, 2014; He et al., 2014; Lu et al., 2015; Cheng et al., 2016; Li et al., 2016; Wang et al., 2018) showed significant effect of AS-IV for decreasing the MI size compared with control group [*n* = 63, SMD −2.58, 95% CI [−3.10 to −2.06], *P* < 0.01; heterogeneity: χ^2^ = 11.81, *df* = 6 (*P* = 0.07); *I*^2^ = 49%] (Figure 3).

#### LVEF

**Figure 4:**
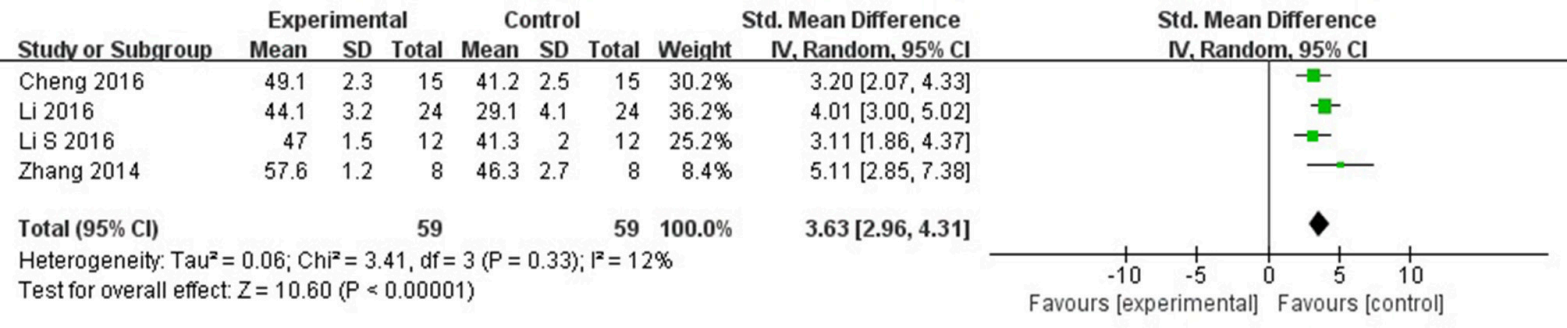
The forest plot: effects of astragaloside IV for increasing left ventricular ejection fraction compared with control group.

Meta-analysis 5 studies (Wang et al., 2012; Zhang et al., 2014; Cheng et al., 2016; Li and Yang, 2016; Li et al., 2016) showed significant effects of AS-IV for improving LVEF compared with control group [*n* = 69, SMD 3.23, 95% CI: 2.17–4.30, *P* < 0.00001; heterogeneity: χ^2^ = 14.62, *df* = 4 (*P* = 0.006), *I*^2^ = 73%]. After removing 1 study (Wang et al., 2012), the animal was treated with AS-IV at 5 weeks after MI other than immediately after establishing the MI model in other studies. Meta-analysis 3 studies (Zhang et al., 2014; Cheng et al., 2016; Li and Yang, 2016; Li et al., 2016) showed significant effects of AS-IV for improving LVEF compared with control group [*n* = 59, SMD 3.63, 95% CI: 2.96–4.31, *P* < 0.0001; heterogeneity: χ^2^ = 3.41, *df* = 3 (*P* = 0.33), *I*^2^ = 12%] (Figure 4). The study (Wang et al., 2012) showed significant effect of AS-IV for improving the LVEF (*P* < 0.05).

#### FS

**Figure 5:**
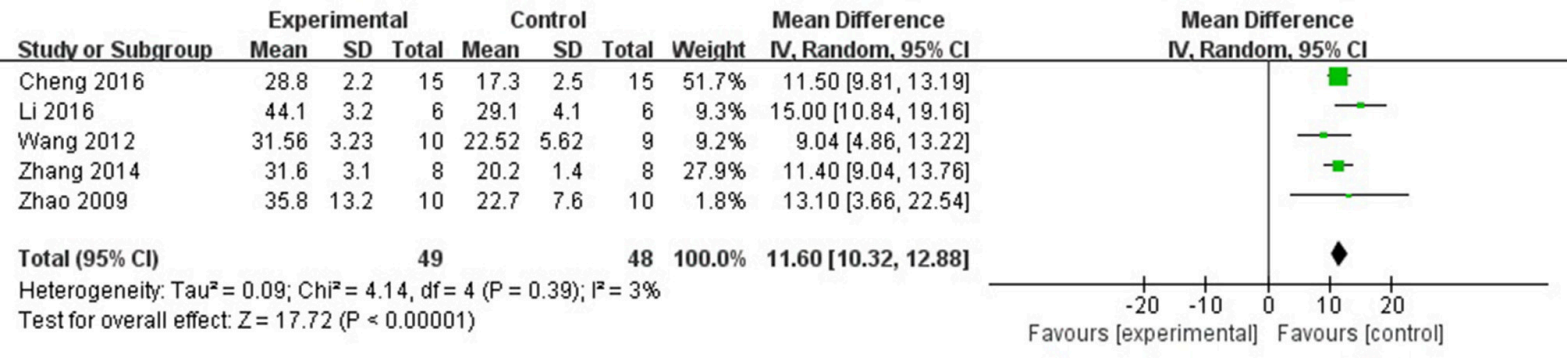
The forest plot: effects of astragaloside IV for increasing shortening fraction compared with control group.

Meta-analysis of 6 studies (Zhao et al., 2009; Wang et al., 2012; Zhang et al., 2014; Cheng et al., 2016; Li and Yang, 2016; Li et al., 2016) showed significant effects of AS-IV for improving FS compared with control group [*n* = 57, MD 10.28, 95% CI: 6.78–13.77, *P* < 0.0001; heterogeneity: χ^2^ = 41.25, *df* = 5 (*P* < 0.0001), *I*^2^ = 88%]. After sensitivity analyses, we removed 1 study (Li and Yang, 2016) that was more than 30% died rate during experiment. Meta-analysis of 4 studies (Zhao et al., 2009; Wang et al., 2012; Zhang et al., 2014; Cheng et al., 2016; Li et al., 2016) showed significant effects of AS-IV for improving FS compared with control group [*n* = 49, MD 11.60, 95% CI: 10.32–12.88, *P* < 0.0001; heterogeneity: χ^2^ = 4.14, *df* = 4 (*P* = 0.39), *I*^2^ = 3%] (Figure 5).

#### The level of ST-segment depression

One study (Liu et al., 2010) reported that AS-IV can improve the ST-segment depression compared with control (*P* < 0.05).

#### Cardiac enzymes and/or cardiac troponin

**Figure 6:**
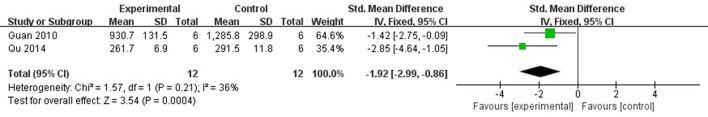
The forest plot: effects of astragaloside IV for decreasing lactate dehydrogenase compared with control group.

Meta-analysis of 2 studies (Guan et al., 2010; Qu et al., 2014) showed significant effect of AS-IV for decreasing the LDH compared with control group [*n* = 12, SMD −1.92, 95% CI [−2.99 to −0.86], *P* < 0.01; heterogeneity: χ^2^ = 1.57, *df* = 1 (*P* = 0.21); *I*^2^ = 36%], (Figure 6). CK, cTnT, and cTnI were reported in one study respectively (Tu et al., 2013; Qu et al., 2014; Wang et al., 2018), they showed that AS-IV had significant effects for reducing CK, cTnT and cTnI compared with control group (*P* < 0.05).

### Cardioprotective mechanisms

**Figure 7:**
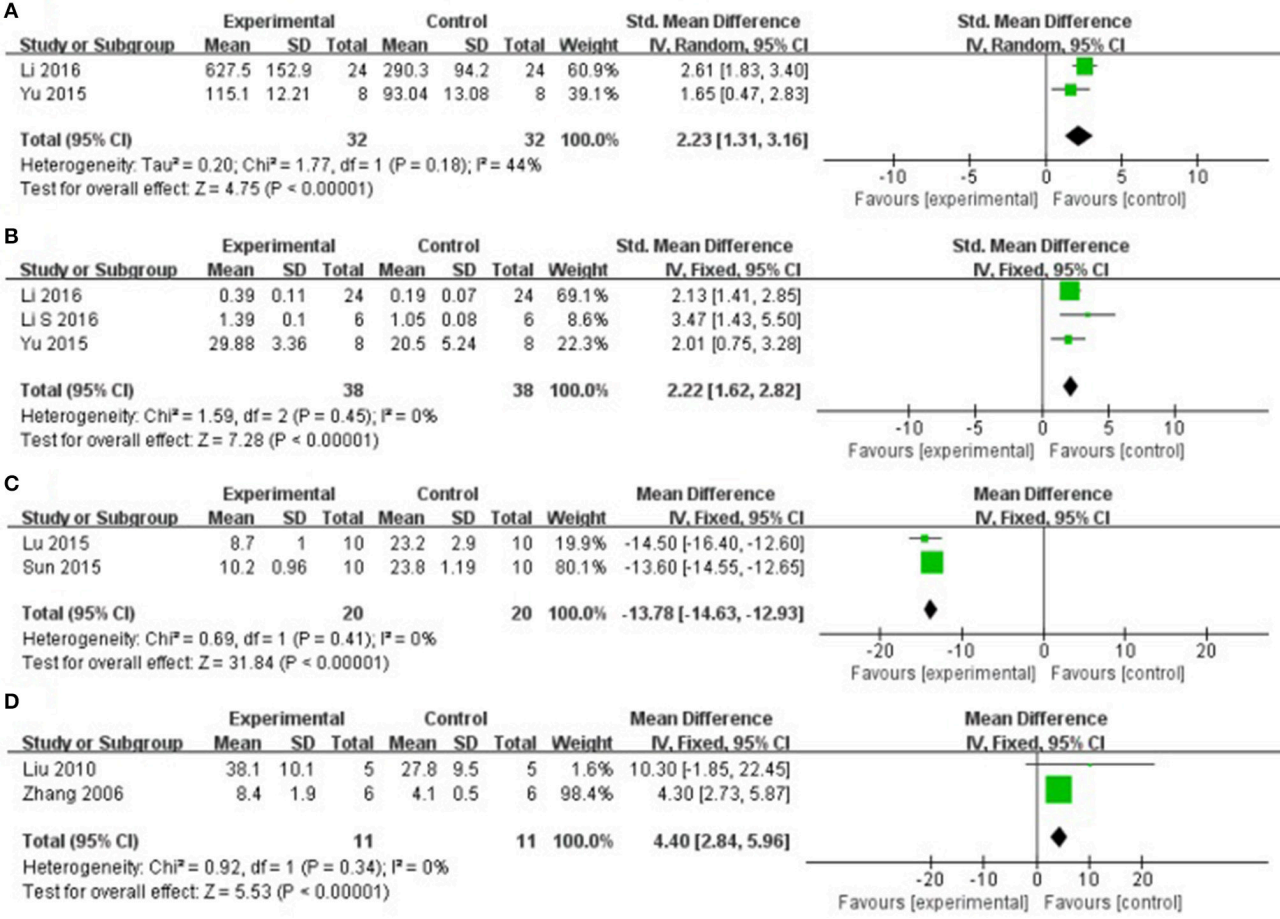
**(A)** The forest plot: effects of astragaloside IV for increasing vascular endothelium growth factor compared with control group. **(B)** The forest plot: effects of astragaloside IV for increasing microvessel density compared with control group. **(C)** The forest plot: effects of astragaloside IV for decreasingmyocardial cell apoptosis rate compared with control group. **(D)** The forest plot: effects of astragaloside IV for increasing coronary blood flow compared with control group.

**Figure 8:**
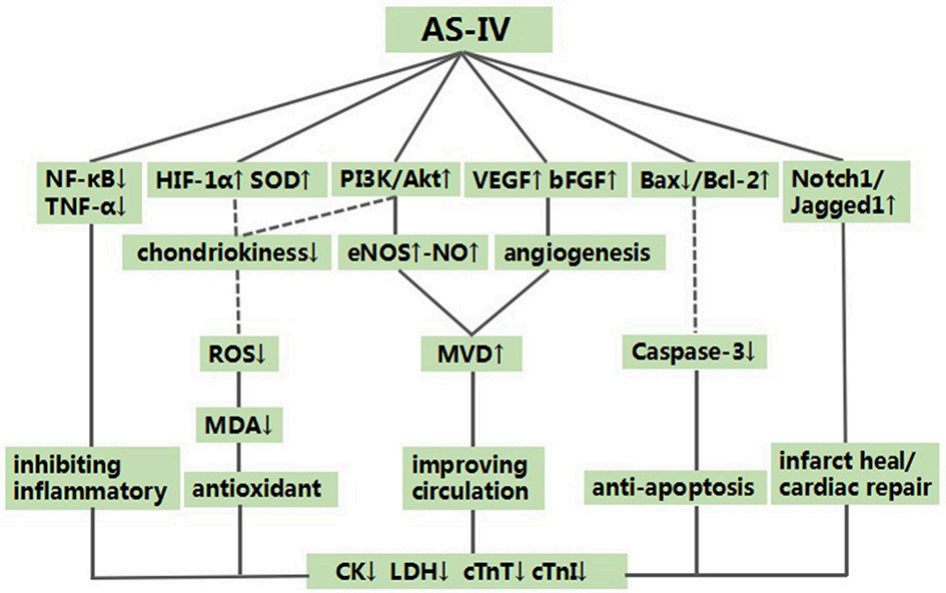
A schematic representation of cardioprotective mechanisms of astragaloside IV for myocardial ischemia/reperfusion injury. Solid lines indicate established effects, whereas dashed lines represent putative mechanisms.

Compared with controls, meta-analysis of 2 studies (Yu et al., 2015; Li et al., 2016) showed that AS-IV significantly increasing VEGF [*n* = 32, SMD 2.23, 95% CI [1.31–3.16], *P* < 0.01; heterogeneity: χ^2^ = 1.77, *df* = 1 (*P* = 0.18); *I*^2^ = 44%], (Figure 7A); 3 studies (Yu et al., 2015; Li and Yang, 2016; Li et al., 2016) for increasing MVD [*n* = 38, SMD 2.22, 95% CI [1.62–2.82], *P* < 0.01; heterogeneity: χ^2^ = 1.59, *df* = 2 (*P* = 0.45); *I*^2^ = 0%], (Figure 7B); 2 studies (Lu et al., 2015; Sun et al., 2015) for reducing myocardial cell apoptosis rate after sensitivity analyses, [*n* = 20, MD −13.78, 95% CI [−14.63 to −12.93], *P* < 0.01; heterogeneity: χ^2^ = 0.69, *df* = 1 (*P* = 0.41); *I*^2^ = 0%], (Figure 7C); 2 studies (Zhang et al., 2005; Liu et al., 2010) showed that AS-IV significantly increasing CBF [*n* = 11, SMD 4.40, 95% CI [2.84–5.96], *P* < 0.01; heterogeneity: χ^2^ = 0.92, *df* = 1 (P = 0.34); *I*^2^ = 0%], (Figure 7D); 1 study (Lu et al., 2015) for decreasing TNF-α (*P* < 0.05); 3 study (Tu et al., 2013; Lu et al., 2015; Cheng et al., 2016) for decreasing NF-κB; 1 study (Yu J. et al., 2017) for increasing HIF-1α; 1 study (Guan et al., 2010) for decreasing SOD, MDA and NO (*P* < 0.05). In addition, 7 studies (Zhao et al., 2009; Tu et al., 2013; Liu and Yi, 2014; Lu et al., 2015; Ma and Wang, 2015; Cheng et al., 2016; Wang et al., 2018) which utilized Bax and Bcl-2 as outcome measure failed for pooling analysis, however they all reported that AS-IV significantly increasing Bcl-2 and decreasing Bax (*P* < 0.05). We summarized a schematic representation of cardioprotective mechanism of AS-IV for myocardial I/R injury (Figure 8).

## Discussion

### Summary of evidence

In this preclinical systematic review, we assessed the efficacy of AS-IV for myocardial I/R injury according to 22 studies with 484 animals. The evidence available from present study revealed that AS-IV exerted potential cardioprotective function in acute MI largely through promoting angiogenesis, improvement of the circulation, antioxidant, anti-apoptosis and anti-inflammatory.

### Limitations

First, no study provided calculation of sample size and blindness of model establishment and outcome measurement that is pesearch. Second, the deficiency of negative studies might lead the effecacy to be overestimated. Thus, the dominance of positive studies might lead the efficacy to be overestimated. Third, MI generally occurs in patients with other complications, such as old age, diabetes, hypertension, and hyperlipidemia (Blankstein et al., 2012); However, only 5 studies (Liu et al., 2010; Qu et al., 2014; Huang et al., 2015; Sun et al., 2015; Yu et al., 2015) selected appropriate animal model. Fourth, 3 studies (Liu et al., 2010; Gong and Sun, 2013; Li et al., 2016) adopted female animals, it cannot be ignored that the heart protection of estrogen has been reported both in clinical and preclinical studies (Menazza et al., 2017).

### Implications

The high-quality preclinical studies are crucial to transform preclinical data to clinic (Ramirez et al., 2017). Thus, we suggest that further design of the studies should refer to the arrival guidelines (Carol et al., 2010) and use appropriate animals, random allocation, blinded induction of model, and blinded assessment of outcomes to improve the accuracy of the results.

The molecular and biological mechanisms of the cardioprorective effects of AS-IV have not been fully elucidated. The present study showed that AS-IV exerted the cardioprorection and the possible mechanisms are summarized as follows: (1) promoting angiogenesis and improving MVD (Yu et al., 2015; Li and Yang, 2016; Li et al., 2016) through increasing the expression of VEGF (Yu et al., 2015; Li et al., 2016) and basic fibroblast growth factor (bFGF) (Yu et al., 2015); (2) inhibition of apoptosis through down-regulating the expression of caspase-3 (Liu and Yi, 2014; Lu et al., 2015; Ma and Wang, 2015; Sun et al., 2015), and increasing the expression of Bcl-2 and reducing the expression of Bax protein in the cardiac myocytes (Zhao et al., 2009; Tu et al., 2013; Liu and Yi, 2014; Lu et al., 2015; Ma and Wang, 2015; Cheng et al., 2016; Wang et al., 2018); (3) improvement of the coronary flow by enhancing the expression of NO via up-regulating the expression of endothelial nitric oxide synthase (eNOS) (Zhang et al., 2005; Liu et al., 2010); (4) upregulating HIF-1α (Yu J. et al., 2017) and enhancing SOD-induced antioxidant via attenuating chondriokinesis to reduce the release of MDA (Zhang et al., 2005; Guan et al., 2010); otherwise, reducing the reactive oxygen species (ROS) to decrease myocardial cell lysis by regulating the PI3K/Akt/mTOR pathway (Zhang et al., 2014); (5) protecting against energy metabolism disorder through reducing the concentration of calcium in cardiac myocytes (Wang et al., 2012, 2018; Tu et al., 2013; Lu et al., 2015; Sun et al., 2015); (6) anti-inflammatory through inhibiting the expression of TNF-α (Lu et al., 2015) and NF-κB (Tu et al., 2013; Lu et al., 2015; Cheng et al., 2016); (7) upregulating Notch1/Jagged1 signaling (Yu J. et al., 2017) which may be involved in infarct healing and cardiac repair (Li et al., 2010; Gude and Sussman, 2012). As mentioned above, cardioprotective mechanism of AS-IV for myocardial I/R injury was largely through promoting angiogenesis, improvement of the circulation, antioxidant, anti-apoptosis and anti-inflammatory.

It is well known that animal experiments have contributed to our understanding of effecacy and mechanisms for diseases (Hackam and Redelmeier, 2006). The present study showed AS-IV significantly decreased the MI size and cardiac enzymes, decreased cardiac troponin and increased the decline degree in ST segment. Therefore, it provides a preclinical evidence-based approach to develop AS-IV for acute MI. However, the translation of preclinical experiment which results in a prediction of the effectiveness of treatment strategies in clinical trials is still challenging (Hackam, 2007). The application of excessive drug doses and the timing of drug administration in animal models, which are inapplicable for human disease, are considered to be two of the main reasons for the failure to translate from animal models to human (Baker et al., 2014). In the present study, doses of AS-IV and timing for initial administration in animal models were inconsistent among the 22 included studies. Thus, we suggest further studies to determinate the optimal gradient doses and timing of administration in animal models of myocardial I/R injury. After that, given the huge gap between the animal studies and the clinical trials, the rigorous RCTs of AS-IV are needed.

## Conclusion

We have provided a first comprehensive systematic review of AS-IV on animal studies and the findings indicate that AS-IV exerted potential cardioprotective function in acute myocardial I/R injury largely through promoting angiogenesis, improvement of the circulation, antioxidant, anti-apoptosis and anti-inflammatory.

## Author contributions

QZ, J-ZZ, X-YB, P-CZ, QT, Y-YH, Q-HZ, and K-JZ designed the study. QZ and J-ZZ collected the data. G-QZ and J-ZZ performed all analyses. QZ, G-QZ, and YW wrote the manuscript. All authors contributed to writing of this manuscript.

### Conflict of interest statement

The authors declare that the research was conducted in the absence of any commercial or financial relationships that could be construed as a potential conflict of interest.
